# Possible relationship between the gut leaky syndrome and musculoskeletal injuries: the important role of gut microbiota as indirect modulator

**DOI:** 10.3934/publichealth.2023049

**Published:** 2023-08-22

**Authors:** Jesús Álvarez-Herms, Adriana González, Francisco Corbi, Iñaki Odriozola, Adrian Odriozola

**Affiliations:** 1 Department of Genetics, Physical Anthropology and Animal Physiology, University of the Basque Country UPV/EHU, 48080 Leioa, Spain; 2 Phymo^®^ Lab, Physiology, and Molecular laboratory, Spain; 3 Institut Nacional d'Educació Física de Catalunya (INEFC), Centre de Lleida, Universitat de Lleida (UdL), Lleida, Spain; 4 Health Department of Basque Government, Donostia-San Sebastián, Spain

**Keywords:** gut microbiota, sports injuries, intestinal permeability, inflammation, nutrition

## Abstract

This article aims to examine the evidence on the relationship between gut microbiota (GM), leaky gut syndrome and musculoskeletal injuries. Musculoskeletal injuries can significantly impair athletic performance, overall health, and quality of life. Emerging evidence suggests that the state of the gut microbiota and the functional intestinal permeability may contribute to injury recovery. Since 2007, a growing field of research has supported the idea that GM exerts an essential role maintaining intestinal homeostasis and organic and systemic health. Leaky gut syndrome is an acquired condition where the intestinal permeability is impaired, and different bacteria and/or toxins enter in the bloodstream, thereby promoting systemic endotoxemia and chronic low-grade inflammation. This systemic condition could indirectly contribute to increased local musculoskeletal inflammation and chronificate injuries and pain, thereby reducing recovery-time and limiting sport performance. Different strategies, including a healthy diet and the intake of pre/probiotics, may contribute to improving and/or restoring gut health, thereby modulating both systemically as local inflammation and pain. Here, we sought to identify critical factors and potential strategies that could positively improve gut microbiota and intestinal health, and reduce the risk of musculoskeletal injuries and its recovery-time and pain. In conclusion, recent evidences indicate that improving gut health has indirect consequences on the musculoskeletal tissue homeostasis and recovery through the direct modulation of systemic inflammation, the immune response and the nociceptive pain.

## 1. Introduction

The musculoskeletal system (bone, cartilage, skeletal muscle, tendon, etc.) enables movement and physical activity. Sports injuries, especially those affecting musculoskeletal structures, are common among endurance athletes [1] because of their mechanical overuse of joint structures [2], tendons [3], and skeletal muscles [4]. These injuries can range from minor sprains to more severe traumatic injuries, further impacting athletic performance and overall health [5]. In addition, injuries could limit sports practice and physical capacities, thereby affecting psychological well-being. On the other hand, the first limiting factor of exercise movement is pain [6],[7], which can be associated with the alteration of nociceptive cells and neuropathic inflammation [8]. While the causes of sports injuries are multifactorial and complex, emerging evidence suggests that a chronic state of systemic inflammation may reduce musculoskeletal functions.

The normal function of intestinal permeability refers to the ability of the intestinal barrier to selectively allow for the passage of luminal pathogens, toxins, antigens, nutrients, and water-electrolytes, while maintaining a barrier function against the entry of harmful substances [9]. In contrast, an impairment of permeability, known as ‘leaky gut syndrome’ [10],[11], is associated with an aberrant immune response [11] and higher inflammatory systemic conditions associated with endotoxemia [12],[13]. During the last few decades, several studies have reported that impaired gut permeability could be related to higher systemic endotoxemia, which promotes a chronic state of inflammation and autoimmunity [10],[11]. This systemic endotoxemia promotes proinflammatory systemic responses, thereby elevating the nociceptive sensitivity to pain [14]. The main causes that promote gut leaky syndrome are related to factors such as diet [15], social or environmental stress [16], intake of medications [17], toxics [18], and intensive physical activity [19]–[22].

Current literature suggests a relationship between leaky gut syndrome and different factors influencing musculoskeletal homeostasis and healing. Among them, nutrition interventions (including pre- and probiotic intake) promote healthy modulation of the gut microbiota (GM) and have been suggested as a promising novel therapeutic intervention to modulate pain and systemic inflammation [23]–[27]. A GM dysbalance has been linked to various intestinal diseases [12] and other systemic conditions [13]. Given the potential role of GM and intestinal permeability in human health and disease, it is reasonable to consider the potential relationship between gut health and new therapeutic treatments for musculoskeletal and joint injuries [28]. The present review focuses on alterations in GM directly affecting intestinal permeability and promoting leaky gut conditions. This intestinal disruption can be associated with systemic endotoxemia, low-grade systemic inflammation, and alterations of the musculoskeletal process of healing and return to homeostasis. Therefore, the main aim of this article is to review the available evidence that suggests the importance of maintaining gut health and GM homeostasis in order to modulate systemic inflammatory and immune responses during the processes of local tissue damage, inflammation and pain. We searched the available literature in Pubmed/Medline, Web of Science, and Sport Discus using the terms “gut, leaky, microbiota, athletes, sport, performance and injury”, and their combinations, trying to provide a balanced view on the potential relationship between intestinal health and the risk of musculoskeletal injuries in athletes. Whenever possible, we tried to propose evidence-based (but also based on the long practical experiences of the authors) strategies to prevent the transition from acute to chronic musculoskeletal pain and injuries. Finally, different gaps in the literature are reported to stimulate future scientific interest in the study of the symbiotic relationship between GM and the host to prevent musculoskeletal injuries in both athletes and the general population.

## 2. The relationship between the gut microbiota and gut permeability: the role of inflammation and pain in adaptive innate immunity

**Figure 1.**
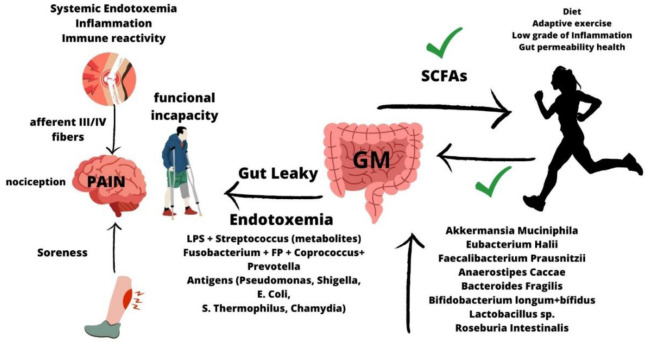
Exercise and diet are two important factors that alter gut microbiota and intestinal permeability. In optimal conditions, adaptive exercise promotes increased positive commensal bacteria related to positive effects on systemic health and better physical performance. In addition, diet can negatively modulate GM and promote gut dysbiosis with aberrant consequences for the intestinal epithelium. Leaky gut syndrome is the condition where selective intestinal permeability is impaired, and positive and negative metabolites, bacteria, and toxins pass to the bloodstream, increasing systemic endotoxemia. In these conditions, the immune response promotes a low-grade systemic inflammation that alters nociception and pain sensitivity, reducing immune adaptive capacities in musculoskeletal tissues and promoting soreness, lack of recovery, and local inflammation. Sports injuries are an important cause of underperformance and physical progression in athletes. Direct consequences are related to overuse during exercise, but indirect factors such as systemic inflammation may contribute to such injuries. In this regard, gut health modulates immune and systemic responses related to inflammation and pain. SCFAs (short chain fatty acids); sp. (species); GM (gut microbiota); LPS (lipopolysaccharides); *E. coli (Escherichia coli); S Thermophilus (Streptococcus Thermophilus*); *FP (Faecalibacterium Praustnitzii)*.

Physical exercise is a determinant factor that can alter gut homeostasis and modulate GM and intestinal permeability [1],[2]. Exercise elevates inflammatory markers post-exercise, both in the intestines and the whole body [28]–[35], thereby increasing the activation of the sympathetic nervous system and causing splanchnic hypoperfusion and intermittent ischemic-reperfusion in the intestines [36]. Ischemia-reperfusion post-exercise elevates intestinal inflammation of the epithelium and disrupts gene response of enterocytes; for example, it induces the chronic activation of HIF-2α with lower HIF-1α expression, promoting this persistent inflammation in the epithelium [37]. Moreover, exercise can aggravate intestinal damage because an elevated body temperature [38] induces a dehydration state [39], which in sum also exacerbates intestinal hypoperfusion, thereby contributing to greater gut leakage permeability. In this context, a higher inflammatory response produced by exercise but also accompanied by systemic endotoxemia produces an underlying over-immune activation of the musculoskeletal structures [3],[40]–[43]. This pro-inflammatory environment promotes higher risks of suffering illnesses and/or musculoskeletal tissues injuries [44].

The GM is the community of microorganisms that inhabit the human digestive tract, including bacteria, fungi, and viruses [45]. These microorganisms are crucial in various physiological processes, including gut homeostasis, immune modulation, metabolism, and gut barrier integrity [46]. The GM is a dynamic ecosystem of microorganisms that are relatively stable during each individual's life [47]. A more balanced GM contributes to average intestinal permeability and systemic health [19]. In this regard, it can exert a potent influence on the normal function of intestinal permeability, which plays a relevant role in developing and recovering from such injuries [48],[49].

High-intensive exercise increases both systemic and local acute inflammation, involving musculoskeletal tissues, and increasing the risk of suffering from sports overload/injuries. In addition, it affects gut homeostasis, and promotes adaptive changes in the GM and intestinal epithelium. Previous studies have reported that athletes presenting a previous dysbiosis of the GM had more significant levels of systemic inflammation and a lower capacity for recovery from intensive exercise [26]. Therefore, when interventions were aimed to improve gut microbiota and reduce leaky gut syndrome, a reduction in the risk of injuries was observed [26]. For example, a study conducted by Jhun et al. [26], reported that administering a probiotic strain of Lactobacillus could slow the progression of osteoarthritis in mice by inhibiting joint pain and any associated inflammation. Until the data is available, no exact mechanisms are understood about the relationship between gut homeostasis and musculoskeletal injuries. However, it is suggested that gut dysbiosis and gut leaky syndrome have a link to higher systemic inflammation and immune dysfunction, which may increase the risk of suffering from musculoskeletal pain and tissue degeneration and damage [50]. In fact, some publications have described a relationship between levels of specific gut bacteria and local inflammation, nociceptive effects, and pain. For example, higher concentrations of Faecalibacterium prausnitzii correlated with antinociceptive effects in a rat model [51]; contrarily, increased levels of the Streptococcus species were associated with knee osteoarthritis (OA) pain [43] and depleted levels of the Coprococcus species depleted were associated with widespread chronic pain [14]. Therefore, the modulation of the body's inflammatory [52] and immune states [24],[49] can alter the nociceptive perceptions and increase response to pain (hyperalgesia) [23],[49].

The intestine exerts a functional barrier with the rest of the body through the epithelium and the mucous layer [53]. In optimal conditions, the gut barrier selectively recognizes nutrients, metabolites, and specific beneficial molecules that pass from the intestinal lumen to the bloodstream [21]. Thus, it prevents the translocation of potentially hostile luminal pathogens, antigens, toxins, and other harmful molecules into the body. Therefore, the intestinal barrier acts as a functionally selective and immunological organ, protecting the internal body from penetrating pathogens, macromolecules, and other toxins [54]. The integrity of normal intestines depends on some components that are in constant regeneration (epithelial cells, the mucosa that covers it, the mucus and the microbiota). The intestinal mucosa is constantly exposed to different biological stimuli, mainly related to commensal and pathogenic bacteria, but also other molecules such as nutrients, toxics, and medication [55]. The intestinal mucus represents the first barrier between the intestinal lumen and the mucosal tissue, where microorganisms strengthen the barrier function and integrity of the intestinal epithelium [56].

The intestinal mucosa comprises mucins (glycoproteins) secreted by the intestinal epithelium goblet cells. This layer protects from proteolysis, prevents the attachment of pathogens to the intestinal epithelium, is a niche for commensal bacteria, and retains molecules such as host defense peptides, IgA, and macronutrients [53]. The enterocytes, which form the epithelium, are interconnected by the so-called tight junctions (TJs), which are intracellular proteins that respond to different stimuli, thereby producing changes in their integrity [57]. TJs are multiprotein complexes composed of identified proteins such as occludins, tricellulin, claudins, junctional adhesion molecule transmembrane proteins, and zonula occludens 1 scaffold proteins [57]. The permeable pass of molecules across the intestinal epithelium can be produced in two ways: 1) transepithelial or transcellular transport (specific passage of solutes through epithelial cells by specialized transporters) and 2) paracellular transport (transport through the spaces between epithelial cells). Paracellular permeability is regulated by TJs and is the main route for the passive flow of water and solutes across the intestinal epithelium.

The GM acts as an endocrine organ, and promotes protective, homeostatic, metabolic, and immune functions within the other parts of the body [58]. Moreover, the GM has a fundamental role in the digestion and absorption of nutrients [22], the production of vitamins [59] and neurotransmitters [60] and the protection against pathogens [61]. Moreover, the microbiota has an essential role in developing and maintaining the intestinal barrier, which contributes to the organism's homeostasis [71] and regulates systemic inflammation [4],[43]. In addition, they play essential roles in the modulation of the oxidative stress through various mechanisms of intestinal absorption of antioxidants [62]. Specifically, some bacterial species such as Lactobacillus plantarum, Lactobacillus gasseri, Lactobacillus fermentum, Lactococcus lactis, Streptococcus thermophilus, Lactococcus, and Bifidobacterium genera have all been shown to elevate intestinal antioxidant levels [63].

The GM ecosystem is highly malleable and rapidly changes according to exposure to intrinsic and extrinsic disruptors such as adverse pathogens, poor nutrition, and different environmental conditions such as hypoxia and/or heat [64]. Thus, interventions that improve the GM ecosystem could rapidly reduce either pain or inflammatory conditions during such conditions or injury processes [27],[65].

Otherwise, the functions of GM are also essential in the maturation of the innate immune system, producing circulating metabolites, hormones, neurotransmitters, cytokines, and developing lymphoid cells [40]. It is essential for the epithelial cells and the mucosal immune system to distinguish pathogenic and non-pathogenic agents to modulate immune responses. Commensal bacteria from intestinal microbiota exert a protective effect on the intestinal barrier function, thereby preventing the colonization of pathogens and interacting with enterocytes through pathogen-associated-molecular-patterns (PAMPs) receptors [66]. Bacteria communicate with immune cells such as macrophages, dendritic cells, and lymphocytes through molecules such as toll-like receptors (TLRs). TLRs alert the immune system to the presence of microbial antigens in PAMPs. For example, the TLR4 receptor recognizes lipopolysaccharides and identifies them as pathogenic. Dysbiosis of the GM increases the number of Gram-negative bacteria in the gut, translocate lipopolysaccharides (LPS) across the inflamed and impaired intestinal permeability, and promotes systemic inflammation and endotoxemia, which finally affects musculoskeletal structures [66],[67].

Figure 1 depicts how exercise and GM modulate systemic and local inflammation by modulating the adaptive immune system and intestinal permeability. Healthy GM contribute to improving physical exercise and protects the intestinal barrier by shielding interactions of commensal bacteria with the mucosa and mucus. Pain sensitivity is altered through the local and systemic levels of inflammation because it activates the immune system and nociceptive receptors. In practice, it is the main limiting factor in exercise and motion

## 3. Gut leaky syndrome, chronic systemic inflammation and musculoskeletal injuries: a new field of study?

Physical exercise can exert potential health benefits for individuals [68]. However, high volumes and/or intensive exercise practice can produce two types of musculoskeletal injuries: 1) mechanical overuse/overload injuries and 2) traumatic injuries [69].

In the last one, a very high force stimulus is applied to the tissue, above its failure strength threshold, causing its injury. In this situation, the relationship between the microbiota and injuries is questionable. On the other hand, in overuse injuries, motor pattern repetition under injury threshold is presented and induces many adaptive and acute inflammatory changes in bones, muscles, tendons, and joint structures. The critical nature of the body's musculoskeletal structures requires constant homeostatic activation of the innate immune system to restore equilibrium [70]. In this regard, most inflammatory responses prepare the body and tissues to fight against stress and other systemic disruptions [71]. In short, acute inflammatory responses can be positive, promoting adaptive mechanisms that restore structures and maintain homeostasis in an optimal range. However, when chronic local and systemic inflammation states co-exist, an over-activation of the immune system can produce aberrant reactivity in tissues and organs [72]. The result of this process is usually pain and loss of motor function, as the body reduces its systemic adaptive capacities to fight against stress, reducing musculoskeletal repair. For example, during aging, the adaptive capacity of the immune system could be impaired by dysbiosis of the GM and leaky gut syndrome [73].

The present work emphasizes the important relationship between systemic inflammation and the higher risk of developing musculoskeletal injuries and impaired recovery and regeneration of connective and muscular structures [3],[42],[74],[75]. In the last few years, different studies have postulated that the GM exerts an important function in the modulation of the immune and inflammatory response in the whole body; however, there is not a concise field of study about the relationship between ‘leaky gut syndrome’ associated with GM dysbiosis and musculoskeletal injuries. Taking into account that literature is scarce on the topic and longitudinal studies do not exist, this study would summarize the main studies that specifically depict the relationship between GM, systemic inflammation, and musculoskeletal injuries.

**Figure 2.**
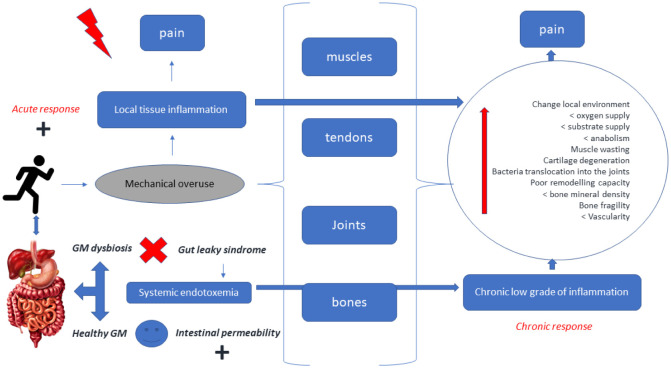
Regular exercise promotes local tissue inflammation of musculoskeletal structures (muscles, tendons, joints, and bones). In some cases, acute inflammation is a positive adaptive response and promotes positive adaptive responses in those tissues. However, if a pro-inflammatory response persists, pain perception appears. Pain is an unpleasant condition for which athletes should reduce their physical practice. Gut health, particularly the gut microbiota, directly communicates with the intestinal epithelium through the mucosa. Leaky gut syndrome promotes systemic endotoxemia and may cause chronic low-grade inflammation. This chronic response aggravates local inflammation in musculoskeletal tissues increasing pain and structural damage, degeneration, fragility, stiffness, poor remodeling, lower anabolism, and oxygen-substrate supply.

### 3.1. Muscles

Muscles are highly malleable, plastic tissues that respond to mechanical and physical loading stimuli. However, it has been reported that the persistence of a chronic proinflammatory state impairs the capacity of satellite cell regeneration and tissue repair [76],[77]. Taking into consideration that athletes cannot eliminate mechanical load and local inflammation is inherent to the sport itself and its specificity, other unresolved systemic disorders may establish systemic inflammatory events and impair muscle performance [78]. In fact, it has been described how the aging process hinders the adaptive process in tissues and the resolution of local inflammation, as characterized by a proinflammatory state [79]. In other chronic pathologies associated with increased systemic inflammation, local muscle involvement has also been demonstrated [80]. Based on the literature, systemic factors influence the cells involved in skeletal muscle healing and growth, thereby slowing the repair process with a reduction in the synthesis and an up-regulation in the degradation of contractile proteins in muscle fibers [81].

Additionally, the gut-muscle axis has been a growing area of research in recent years [82]–[84]. It was reported that the GM influences muscular function and modulates the cycle of anabolism/catabolism [82]. In mice, increasing bacteria such as Eubacterium rectale, Lactobacillus plantarum TWK10, or Clostridium coccoides [85] seems to positively increase muscular function. Contrarily, Nay et al. [86] confirmed that treatment with antibiotics impairs the endurance running performance and muscle fatigability of mice. In humans, a study reported that older people with higher muscular performance presented high levels of genus Prevotella and Barnesiella [87].

Longer and more intense exercise loads increase the risk of producing muscle injuries [88]. Moreover, other systemic conditions associated with chronic inflammation can aggravate muscle symptoms such as soreness or injuries [74],[80]. As previously mentioned, a disbalanced state of the GM can favor ‘leaky gut syndrome’, thereby increasing levels of systemic endotoxemia and a state of low-grade chronic inflammation [10],[11]. Muscle wasting, understood as a catabolic state of the tissue, can be caused by the local inflammation promoted by the exercise itself and moreover by an inadequate systemic inflammatory state [89]. Under these conditions, the environment where muscle tissue can regenerate, obtain nutrients, or oxygenate itself would deteriorate. These systemic conditions can end up deteriorating muscle recovery, regeneration, and anabolism processes, thereby favoring a greater risk of injury, acute muscle soreness, or pain [88],[89]. In addition, chronic inflammation induces loss of muscle mass, a lack of regenerative capacity, and deterioration of oxygenation and metabolism processes, influencing tissue health [74].

As we reported, an acute inflammatory response in muscles is usually coupled with regeneration and repair following injury [88] and exercise [90]. After exercise, the inflammatory response is crucial for repairing structural damage and for stimulating muscle protein synthesis [91],[92]. Indeed, it has been shown that accelerated recovery from damage is related to over-expressing IGF-I within skeletal muscle [93]. In this regard, it has been reported that the GM contributes to elevating circulation levels of IGF-I [94]. The overall beneficial or detrimental effect on the muscle function of inflammatory processes is mainly regulated by the tissue response's magnitude related to the previous history of muscle injuries, the systemic and local inflammatory state, and the metabolic and hormonal response [88]. High intensity exercises could influence the persistence of inflammation maintained in muscle tissue due to overuse. The anti-inflammatory capacity of athletes can restore these conditions when there is no exponential increase in sustained systemic inflammation [88].

A local and systemic inflammatory state has been shown to decrease insulin sensitivity and glucose supply to the muscle [95]. In this aspect, the relationship between microbiota-muscle may be an essential field of study to help reduce the chronic inflammatory state in athletes and improve their muscle function [96].

It was shown how the composition of the GM affects muscle function, and how certain bacteria increase under optimal conditions of muscle anabolism (Faecalibacterium Prausnitzii, Roseburia Hominis, Akkermansia, Bifidobacterium and Lactobacillus spp.), unlike others, which were described with sarcopenia (Escherichia Shigella, reducing bacteria and other opportunistic pathogenic bacteria such as Citrobacter, Freundii, Enterococcus faecalis, Campylobacter, Helicobacter, and Staphylococcus Aureus) [95].

A dysbiosis of the GM associated with gut barrier dysfunction increases proinflammatory cytokines, oxidative stress [97] and impairs muscle function and repair [82],[86],[95], while SCFA-producing bacteria (acetate, propionate, and butyrate) promote intestinal homeostasis. In this regard, Karl et al. showed how worsening intestinal homeostasis decreased the muscle function and adaptive capacity in soldiers with high physiological stress [98]. On the contrary, favourable GM increases levels of SCFAs, which act as hepatic substrates in gluconeogenesis pathways [99], and ultimately as substrates (mainly acetate) that reach the muscle.

### 3.2. Tendons

Tendinopathies are the overuse mechanical injuries with the highest proportion among total musculoskeletal injuries (30–50%) [100], and they are associated with chronic tissue inflammation, degeneration, and high grade of afferent sensitivity and pain. In a tendon injury, a programmed (genetic) healing process includes firstly an acute inflammatory period between 1 to 5 days, followed by a subsequent period of fibroblastic proliferation (between 6 days and 6 months post-injury), and finally a collagen remodelling process that takes place between 6 to 12 months post-injury [100].

Tendons require constant remodeling to maintain their tissue architecture and strength levels [100]. Although their structural overuse can cause tendinopathy, chronic systemic and low-grade inflammation resulting from endotoxemia could be another possible trigger for some forms of chronic inflammatory injuries [3],[100]. A chronic state of systemic inflammation has been associated with impaired gut health, which may drive the onset and progression of tissue damage [101]. Thus, gut leakage permeability is a significant risk factor that increases the translocation of pathogenic molecules, thereby exacerbating inflammatory responses in tendon structures. This healing process could be potentially perturbed if systemic inflammatory conditions exist. However, currently limited evidence exists to support or reject this hypothesis [102].

Previous studies have reported how the gut microbiota state correlates with tendon injury regeneration and the healing process [103]. In mice, it was observed how changes in the microbiome influence tendon healing through different strategies, such as mechanical load [104] or medication [105]. These studies concluded that immunomodulatory changes associated with the GM may promote positive tendon healing. Therefore, the modulation of systemic endotoxemia may be an important mechanism to reduce local inflammation and pain in tendon injuries.

### 3.3. Joint structures

Joint structures are affected by many factors, such as mechanical overloads, age, gender, intensity and volume, increasing the risk of degeneration and the lack of healing during inflammatory processes. Osteoarthritis (OA) is a degenerative joint disease with a prevalence in adults of 20% that increases with aging up to 50% [106]. The degeneration of cartilage and bone is present in joint structures and is accompanied by chronic synovial membrane inflammation. Chronic inflammation is present in all joint degenerative processes [14],[107], exacerbating the immune response and favoring OA progression [107]. In recent years, different studies have reported a direct relationship between the GM and the progression of OA [66],[108]. Thus, the progression of joint degeneration could be influenced by GM's dysbiosis and leaky gut syndrome [43],[109],[110]. Leaky gut syndrome promotes the increase of LPS into the bloodstream, promoting endotoxic factors, and synovial fluid triggers a proinflammatory response, affecting articular cartilage destruction [66]. Boer et al. [43] showed that the elevation of intestinal microbiome β-diversity was significantly associated with knee pain and increased Streptococcus spp. The metabolites produced by Streptococcus can activate macrophages via TLR and promote higher inflammation and pain sensitivity in joint structures [43]. Moreover, Clostridium species have been hypothesized to promote sharp pain in OA conditions and, jointly with Streptococcus and other species, activate local or systemic macrophages [10] associated with the serum and synovial presence of LPS-blinding proteins and pain [66]. Other studies reported an elevation of Fusobacteium and Faecalibacterium and a reduction of Ruminococcus in OA [111]. Fusobacterium spp. has also been reported to be elevated in different metabolic diseases [112],[113], while Ruminococcus seems to have protective effects to prevent gut permeability and reduce low-grade chronic inflammation due to its an SCFA's production [114]. The elevation of circulatory LPS in the blood has been related to higher proportions of Escherichia/Shigella, Klebsiella, and Citrobacter species in the GM [115]. Wei et al. [116] reported that higher systemic inflammation was related to more symptomatic pain symptoms and a specific low abundance of positive commensal bacteria such as Roseburia but a high abundance for Desulfovibrio and Biophila. On the other hand, the elevation of bacteria such as Clostridia and Staphylococcus was related to higher OA symptoms, which decreased after 12 weeks of supplementation with green-lipped mussel (GLM) extract or glucosamine sulphate [117]. Therefore, it has been confirmed that the OA condition is related to the abundance of species such as Clostridium spp. and relative decreases for positive homeostatic bacteria such as Bifidobacterium longum and Faecalibacterium prausnitzii [118].

Bacterial antigens (Pseudomonas sp., Shigella spp., Escherichia coli, Streptococcus thermophilus, Chlamydia trachomatis, and Chlamydia pneumonia) within the synovial fluid demonstrate the translocation of intestinal bacteria into the joints [119].

Traditional treatments for joint injuries include the administration of medication to modulate the inflammatory processes. However, the chronic and/or regular consumption of analgesics and anti-inflammatory medications promotes adverse effects on gut health, thereby impairing GM balance [120]. In this regard, nutritional interventions that increase GM diversity may influence joint disease progression and/or healing [43],[109]–[111],[121]. Prebiotics and probiotics provide a promising field of study to modulate the positive functions of the GM and improve the prognosis for musculoskeletal injuries. Prebiotics can promote the growth of certain bacteria, such as those promoting the synthesis of SCFAs, peptide hormones, or neurotransmitters [40]. For example, in obese mice, oligofructose supplementation mitigated OA progression due to the increase of anti-inflammatory species such as Bifidobacterium pseudolongum and other proinflammatory such as Peptococcaceae and Peptostreptococcaceae [122]. Rios et al. [123] confirmed that supplementation with oligofructose and exercise delayed OA development and increased positive bacteria such as Bifidobacterium and Roseburia. The intake of Lactobacillus casei could also act as a potent modulator of pain, inflammatory responses, and cartilage regeneration in the treatment of OA [124]. Another study showed that the administration of Bacillus coagulans (2 billion CFU/day) plus green tea extract and a cocktail of vitamins and minerals (including vitamins A, B, C, D, E, folic acid, and selenium) can reduce joint pain [125].

### 3.4. Bones

In all bone tissues, the acute inflammatory response is positive and enhances the regulated processes of remodeling, healing, and regeneration. Bone remodeling depends on the balance between the actions of osteoblasts, osteocytes, and osteoclast. However, under conditions of proinflammatory chronicity, it can be detrimental to healing [126]. This process happens because there is resorption and suppression of bone formation, with a crosstalk between inflammatory cells (polymorphonuclear leukocytes and cells of the monocyte-macrophage-osteoclast lineage) and cells related to bone healing (cells of the mesenchymal stem cell-osteoblast lineage and vascular lineage) [127].

In a normal condition, regular exercise with mechanical impact loads increases musculoskeletal stress and promotes bone microdamage [128]. Stress fractures are suffered by athletes due to reduced bone mass and a lack of equilibrium between bone formation and resorption [129]. Bone homeostasis is related to vitamin D levels, the capacity of calcium absorption, and other anabolic hormones such as estrogens [129]. During the last decade, emerging evidence has talked about the interaction between the specific gut microbiota bacteria and bone health [130]. In this regard, maintaining a healthy gut microbiota could be crucial to guaranteeing bone homeostasis, regulating sex hormones, vitamin D levels, and calcium absorption [42]. Thus, a decreased GM diversity impairs estrogen production [132] and promotes excessive reabsorption [131]. In mice, the intake of probiotics demonstrated how different Lactobacillus species [42], Bifidobacterium longum [132], and Lactobacillus reuteri [133] seem to be effective in the treatment of bone loss. In contrast, a poor bone remodeling capacity predisposes to degeneration, favoring the risk of stress fractures [128]. In addition, it has been reported that leaky gut syndrome impairs bone health because it reduces bone mineral density [26],[42],[134].

The intake of nutritional prebiotics and supplemental probiotics has been proven effective in preventing bone loss and improving recovery after fractures [135]. The intake of undigestible fiber as oligo- and polysaccharides increases gut fermentation in the colon and promotes SCFA's formation, mainly butyrate from the firmicutes and acetate and propionate from the Bacteroidetes [136]. In the colon, increasing SCFA reduces intestinal pH, thereby improving calcium and phosphate absorption in the intestines [137]. The increased SCFA levels from the GM increase bone formation through higher levels of circulating IGF-1 [138]. The increase of species such as Bifidobacterium and Clostridium has been reported to increase calcium absorption [137], and probiotics containing Lactobacillus and Bifidobacterium are positive for osteoblast activity [137]. Other studies reported how patients with osteoporosis presented abundant species such as Blautia, Actinobacillus, Oscillospira, Bacteroides, and Phascolarctobacterium, whereas Veillonellaceae, Collinsella, and Ruminococcaceae all negatively correlate with the bone disease [139]. Studies with animals and humans have reported that the gut leaky condition [140] increases systemic endotoxemia and promotes a chronic, low grade systemic inflammation, which predisposes subjects to decreased bone reabsorption [134], bone loss, lower bone mineral density, lower hip strength, and bone fragility [141]. Finally, Reinke et al. [142] showed that GM dysbiosis could increase the activation of the immune system and reduce bone fracture healing. In synthesis, gut health, consisting of maintaining a healthy microbiome and effective intestinal permeability, is essential for maintaining bone homeostasis, remodelling, and healing functions [42].

## 4. Could musculoskeletal and joint pain sensitivity be modified through nutrition and GM balance?

As this review noted, gut health is related to microbiota and intestinal permeability and can influence sports injuries' development and/or recovery [21]. Therefore, changing habits that modulate gut health can be easily included in sports routines for improving sports injury prognosis. In addition, lifestyle factors, including nutrition and specific interventions such as intermittent fasting, exposure to toxins and medication, ultra-processed food (additives), exercise training, and environmental and social relations, seem to influence the gut microbiota profile and the systemic health related to the locomotor structures [143].

It was reported that well-trained athletes present a higher diversity and abundance of GM than sedentary or sick subjects [144]–[148]. Athletes are advised to eat four or five times per day and even during exercise, which can be considered an overload stimulus for the intestines, forcing them to work regularly during digestion. In this regard, based on simple carbohydrates, such diets may promote impaired mucosal barrier integrity, thereby elevating gut and systematic tissue inflammation, as shown in musculoskeletal injuries [64]. It was reported that nutritional interventions that decrease intestinal inflammation and modulate healthy GM could be considered therapeutic to promote peripheric tissue repair and joint structures [149],[150]. Some studies in animals and humans have reported that probiotic strains, mainly Lactobacillus and Bifidobacterium, positively modify the GM ecosystem and contribute to regulating effective immune responses, maintaining healthy intestinal permeability, and exerting beneficial effects on bone health, joint structures, and tendons. These probiotics contribute to restoring average gut permeability, which may reduce systemic inflammation, and improve metabolic functions through the administration of butyrate [151] and the reduction of sarcopenia with the Lactobacillus group [152]–[154] (helveticus, casei shirota). Furthermore, specific bacteria groups have been reported to improve the synthesis, digestion, and absorption of amino acids (Fusobacterium, Bacteroides, Veillonella, Megasphaera elsdenii, Selenomonas ruminantium) [155] and de novo synthesis (Streptococcus bovis and Prevotella bryantii) [156].

Pain is a primary factor limiting physical performance, reducing regular exercise practice, and impairing athletes' moods. Pain is an unpleasant sensation related to tissue inflammation and damage [8] that promotes the reduction of physical and perceptive potential as an innate neural mechanism to prevent and protect against more damage. A high grade of local and systemic inflammation is usually related to the elevation of nociceptive pain perceptions [157]. In this way, gut dysbiosis and impaired intestinal permeability increase the systemic level of inflammation, where the immune system reacts as a protective mechanisms increasing pain sensitivity (hyperalgesia) [158],[159]. In this context, the gut is a critical modulator of the onset and development of neurogenic pain and inflammation [24],[160]. Several publications have argued how higher levels of Faecalibacterium Prausnitzii [161] and Coprococcus [14] increase antinociceptive sensations, while others, such as Streptococcus [43] are associated with more severe pain in pathologies such as OA.

It has been hypothesized that positive daily routines, including specific diet interventions, may reduce systemic inflammation and promote a lower activation of nociceptors signaling pain [23]. In this way, a positive elevation of commensal bacteria produces elevated levels of specific molecules and metabolites (SCFAs), modulating inflammation and pain sensitivity in the gut and whole body [162]. These molecules have essential anti-inflammatory properties that are key to gut immunomodulation [163],[164]. The interaction between some nutrients and GM, including polyamines, polyunsaturated fats, Omega 3, polyphenols, flavonoids, and some vitamins and minerals, has been demonstrated to be adequate for modulating pain. Polyamines are naturally present in legumes, soybeans, cereals, mushrooms, and algae, containing particles of spermidine, spermine, and putrescine, which are antinociceptive substances [165]. Polyunsaturated Omega 3 are considered as anti-inflammatory and antinociceptive molecules [25],[166] with analgesic properties in joint pain [167]. Plant-derived polyphenols present in red wine, straw and blueberries, green tea, soy, and the Zinziberaceae family (zerumbone and curcumin) have anti-inflammatory properties [168] regulating the decrease of proinflammatory cytokines TNF-α, IL-6, and IL-1β [169]. Flavonoids such as quercetin in red onions, berries, broccoli, and apples promote antinociception [170]. Curcumin has positively affected chronic musculoskeletal pain and osteoarthritis [171]. Other components, such as nutrients with high doses of selenium [172], magnesium [173], vitamin D supplementation [174] and taurine [175] have been reported as pain modulators, too.

Additionally, some neurotransmitters and hormones synthesized in the gut are essential for modulating neural pain and mood. Directly, GM synthesizes high proportions of total neurotransmitters in the body. Dopamine regulates inflammation and the immune response and it's a crucial modulator of chronic pain and behavior [176]. Serotonin is an essential mood modulator that is synthesized in about 90% in the gut. Strain bacteria such as Clostridia, Bacteroides, and Escherichia can alter serotonin levels, affecting tryptophan production [52]. Other neurotransmitters such as noradrenaline, glutamate, and GABA produced in the gut are related to pain and inflammation, and their production depends on different bacteria such as Lactobacillus, Bifidobacterium, and Bacteroides [52].

Intestinal hormones such as ghrelin and leptin have anti-inflammatory properties that participate in nociceptive regulation and immunometabolic inflammation, which are characteristics of chronic pain conditions [52]. SCFA production levels relate to the modulation of intestinal hormones such as leptin and ghrelin [177]. Ghrelin has anti-inflammatory properties because it increases the level of anti-inflammatory cytokines in the serum and mediates pain [178]. Moreover, leptin modulates neuropathic pain and may be a promising biomarker for predicting acute pain transition to chronic [179]. Therefore, the production of ghrelin and leptin relates to the levels of SCFAs in the gut [177].

Cortisol is the key stress hormone in humans and influences the GM composition [180]. While it does not affect pain, chronic elevation of cortisol impairs the body's capacity to cope with stress [181]. In addition, if stress is maintained chronically, pain perception levels increase due to the maladaptive capacity to manage the inflammatory response [52].

## 5. What are the bacteria related to intestinal permeability and musculoskeletal injuries?

Several studies have shown how some pathologic bacteria may be related to leaky gut syndrome. However, most of them are observational studies in which it's not proven that there's a direct cause-and-effect relationship between bacteria and leaky gut syndrome. For example, a significant positive association was found between the presence of microorganisms such as Fusobacterium or Prevotella and the risk of musculoskeletal injuries [110],[182]. Likewise, some bacterial genera, such as Firmicutes [19] and Escherichia coli [19],[183], were found in greater abundance in individuals with excellent intestinal permeability. Additionally, it was observed that Ruminococcus gnavus degrades the mucus, inducing the translocation of LPS through the intestinal epithelium and increasing leaky gut syndrome [184]. Similarly, the SpvB Salmonella effector redistributes the tight junction proteins (occludin and claudin-1), alters the integrity of the intestinal barrier and facilitates Salmonella dissemination [185]. Table 1 shows some of the most notable mucolytic bacteria associated with intestinal protective functions for permeability and GM balance.

## 6. What are the limitations of the current evidence on the relationship between gut microbiota, intestinal permeability, and musculoskeletal injuries?

There are several limitations to the current evidence on the relationship between gut microbiota, leaky gut syndrome and musculoskeletal injuries. First, most of the studies are observational, which limits the ability to establish causality. Further randomized controlled trials are needed to confirm the findings of the observational studies. Second, the studies are heterogeneous regarding the participants studied, the interventions examined, and the outcomes measured, making it difficult to compare the results across studies. Third, based on different exercise practices and nutritional routines, it is unclear if the findings included here are generalizable to elite, recreational athletes or general populations. Fourth, the mechanisms underlying the relationship between gut microbiota, intestinal permeability, and sports injuries are not fully understood.

However, we hypothesize that adaptive innate immunity related to the intestinal epithelium and the modulation of the inflammatory response could be essential in managing systemic and local inflammation. Finally, most musculoskeletal injuries are disabling, mainly because the neural pain related to the inflammatory processes impairs movement. Furthermore, it is essential to consider that the microbiota and intestinal permeability are complex and influenced by many factors; therefore, it is difficult to determine the influence of a single bacterium or group of bacteria on these processes.

## 7. What are the most effective strategies for preventing and recovering from sports injuries regarding gut microbiota and intestinal permeability?

**Figure 3.**
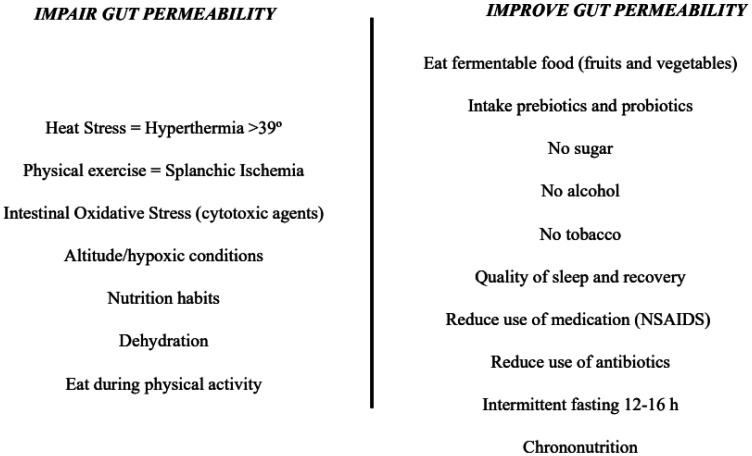
Interventions that can impair/improve gut permeability.

Based on the evidence from the studies included in this review, many factors could be highlighted in the accentuation of intestinal permeability (see Figure 3):

1) Heat stress related to exercise increased core temperatures (hyperthermia> 39ºC), damaging the epithelial cells due to a more outstanding contribution of blood flow to the cutaneous vascular network [186]. Common heat stroke and exertional heat stroke have been associated with gastrointestinal barrier integrity loss and microbial translocation in animal models [187],[188], and indirectly in humans by the presence in blood of microbial products such as endotoxin, D-glucan and procalcitonin [189].

2) Stress due to splanchnic ischemia (cellular hypoxia) generates more significant redistribution of blood flow to the active muscles and peripheral organs (heart and lungs), not reestablishing perfusion until the end of physical activity. The degree of damage produced in the mucosa (surface or deeper layers) depends on the severity of the ischemia and its duration (edema and bleeding, among others) [36]. Similarly, it causes phosphorylation of specific tight junction proteins, promoting enterocyte injury and increased intestinal barrier integrity, and therefore microbial translocation, especially in endurance high-intensity exercise [190]

3) Oxidative stress (tissue damage) production by the action of intestinal cytotoxic agents (oxygen-free radicals, nitric oxide, and cytosines, among others). Regular exercise is associated with an acute increase of oxidative stress and a positive long-term effect on the gut anti-inflammatory response [191]. Similarly, in animal models, acute exercise increased oxidative stress but was not associated with intestinal inflammation [192]. On the contrary, in the exercise overload context, it is essential to note that during the severe splanchnic ischemia and reperfusion phases, many free radicals are created, thereby aggravating the damage to the intestinal mucosa [193].

4) Exercise in altitude or hypoxic conditions has been reported that increase gut permeability and intestinal ischemia more than the same activity performed in normoxia. Moreover, in altitude conditions, the systemic conditions achieved from the environmental conditions increase fluid loss, metabolic stress, and the activation of the sympathetic neural system [194].

5) For example, chronic high-intensity exercise in cyclist competition of three weeks or ultra-endurance running events may increase gut stress due to intestinal ischemia, changes in the intestinal pH, dehydration, hyperthermia, and systemic inflammation [22],[195].

Based on current evidence, promoting a healthy GM can directly improve gut health and intestinal permeability. The better modulation of immune response in the gut reduces systemic and local chronic inflammation, indirectly benefiting musculoskeletal homeostasis. Moreover, has been suggested that interventions on GM may effectively prevent the transition from acute to chronic musculoskeletal pain [196]. Accessible practices to improve gut microbiota diversity and intestinal permeability relate to nutrition and daily routines (see Figure 3).

Easy practices to improve GM diversity and intestinal permeability have relation with the nutrition and daily routines. Among others, they should promote a high microbiota diversity through the intake of prebiotics [197] and probiotics [61]. The reduction of ultra-processed food [61] and non-steroidal anti-inflammatory drugs (NSAIDs) [17] should be taken to avoid excessive antibiotics ingestion, since these can alter the microbiota and increase intestinal permeability [198]. In addition, one should consume probiotic foods, potentially through a specific strain of bacteria or with natural food probiotics such as yogurt and kefir, which contain live strains of bacteria beneficial to the microbiota. In the general population, one should maintain an active and healthy lifestyle since regular physical exercise can improve the microbiota and intestinal permeability [199]. Additionally, one should avoid stress and insufficient sleep, as they can negatively affect the microbiota and intestinal permeability [16]. Adopting stress-management strategies, such as meditation, yoga, or exercise, may help to reduce stress levels and improve gut health. One should incorporate rest and recovery periods into training regimens: proper rest and recovery are essential for maintaining gut health and reducing the risk of musculoskeletal injuries. In addition, massages and other active recovery techniques can help reduce muscle damage and inflammation; therefore, they could be important for gut health. One should include intermittent fasting for 12-16h to improve metabolic efficiency, reduce inflammation and contribute to gut microbiota regulation [150]. Additionally, one should promote the chrono-nutrition regulating meal time to improve endocrine and gut microbiota processes favoring not eating during and immediately post-exercise [200].

## 8. Conclusions

In conclusion, emerging evidence suggests that GM and intestinal permeability may play an essential role in the adaptive innate immunity of the gut, the modulation of systemic and local inflammation, neural sensitivity to pain, and the degeneration of musculoskeletal and joint structures related to sports injuries. Furthermore, the literature suggests that gut microbiota-related gut leaky alterations may decrease the ability to recover and/or repair musculoskeletal tissues after acute damage, inflammation, and injury. Besides, it seems systemic endotoxemia promotes low-grade inflammation that reduces the adaptive capacities in tissues and the immune system, as in aging.

Therefore, athletes must consider maintaining a healthy microbiota and adequate intestinal permeability to reduce the risk of suffering more musculoskeletal injuries related to inflammation and pain. In general population, musculoskeletal non-traumatic injuries specially associated with degenerative processes as osteoarthritis, osteoporosis, tendinopaties or muscular sarcopenia are also related to systemic low-grade of inflammation. To prevent the transition to acute to chronic pain effective interventions based in modulate immunomodulation could be further explored in the follow years.

To this purpose, practical recommendations can be stated, such as eating a diet rich in prebiotics, consuming probiotics, avoiding excessive consumption of processed and sugary foods, maintaining an active lifestyle and healthy, managing stress levels, incorporating rest and recovery periods into training regimens, sleeping sufficiently, and avoid overuse of antibiotics, ultra-processed foods including sport supplements and NSAIDs.

It is important to note that the relationship between gut microbiota, intestinal permeability, and musculoskeletal injuries is complex and still not fully understood. Therefore, further research is needed to determine the mechanisms and specific bacteria underlying this relationship and to develop effective strategies for preventing, managing, and recovering from sports injuries in athletes and general population. In addition, future studies should consider other potential factors that may affect the relationship between gut microbiota, intestinal permeability, and musculoskeletal injuries, such as physiopatological condition, age, gender, and type of activities or sport. Moreover, the relationship between systemic inflammation and hypothalamic sensitivity to pain may have a relation to intestinal endotoxemia and the alteration of nociceptive tolerance and threshold.

In conclusion, the modulation of pain and musculoskeletal injuries could have a new field of treatments based on the intervention with GM and intestinal permeability. In such cases, the analysis of the GM both for athletes as also in general populations, is everyday more the present to improve the individual measurement of the gut health. Even more, the symbiotic relationship between the GM and the host is key to maintaining gut and systemic health. The modulation of inflammatory and immune processes after local stress is very important to regulate acute responses related to musculoskeletal homeostasis. Therefore, reduce possibilities to achieve chronical musculoskeletal states of inflammation, degeneration and pain. According to the specific literature in the topic, local and systemic inflammation is related to the leaky gut syndrome, immune system over-activation, and pain sensitivity.

## Use of AI tools declaration

The authors declare they have not used Artificial Intelligence (AI) tools in the creation of this article.
